# Gene expression patterns and dynamics of the colonization of common bean (*Phaseolus vulgaris* L.) by highly virulent and weakly virulent strains of *Fusarium oxysporum*

**DOI:** 10.3389/fmicb.2015.00234

**Published:** 2015-04-02

**Authors:** Jonathan Niño-Sánchez, Vega Tello, Virginia Casado-del Castillo, Michael R. Thon, Ernesto P. Benito, José María Díaz-Mínguez

**Affiliations:** Departamento de Microbiología y Genética, Instituto Hispano-Luso de Investigaciones Agrarias, Universidad de SalamancaSalamanca, Spain

**Keywords:** *Fusarium oxysporum*, *Phaseolus vulgaris*, confocal microscopy, virulence, effector

## Abstract

The dynamics of root and hypocotyl colonization, and the gene expression patterns of several fungal virulence factors and plant defense factors have been analyzed and compared in the interaction of two *Fusarium oxysporum* f. sp. *phaseoli* strains displaying clear differences in virulence, with a susceptible common bean cultivar. The growth of the two strains on the root surface and the colonization of the root was quantitatively similar although the highly virulent (HV) strain was more efficient reaching the central root cylinder. The main differences between both strains were found in the temporal and spatial dynamics of crown root and hypocotyl colonization. The increase of fungal biomass in the crown root was considerably larger for the HV strain, which, after an initial stage of global colonization of both the vascular cylinder and the parenchymal cells, restricted its growth to the newly differentiated xylem vessels. The weakly virulent (WV) strain was a much slower and less efficient colonizer of the xylem vessels, showing also growth in the intercellular spaces of the parenchyma. Most of the virulence genes analyzed showed similar expression patterns in both strains, except *SIX1*, *SIX6* and the gene encoding the transcription factor FTF1, which were highly upregulated in root crown and hypocotyl. The response induced in the infected plant showed interesting differences for both strains. The WV strain induced an early and strong transcription of the *PR1* gene, involved in SAR response, while the HV strain preferentially induced the early expression of the ethylene responsive factor *ERF2*.

## Introduction

*Fusarium oxysporum* Schlechtend.:Fr. is an anamorphic species complex (FOSC) with considerable morphological and physiological variation (O’Donnell et al., 2009). This fungus is ubiquitous in soils worldwide being able to grow saprophytically or colonizing plants. The pathogenic strains collectively may infect more than 100 different hosts, ranging from globally important crops such as cotton, tomato, banana, and several legumes to immunocompromised patients (Nucci and Anaissie, 2007; Michielse and Rep, 2009). Despite the broad host range at the species 

complex level, the individual isolates are able to infect only one or a few plant species. This allows for a classification into formae speciales based on host-specificity (Booth, 1971; Armstrong and Armstrong, 1975), making the *F. oxysporum* species complex a very attractive system for the study of the relationships between pathogenicity, virulence and host-specificity. *F. oxysporum* f. sp. *phaseoli* infects some species of the genus *Phaseolus*, mainly *Phaseolus vulgaris* and *P. coccineus* (de Vega-Bartol et al., 2011), and is one of the principal agents limiting dry bean production worldwide.

### Pathogenicity Tests

Inoculation of *P. vulgaris* L. cv Blanca Riñón with conidia from the *F. oxysporum* strains tested was mainly carried out as previously described (Alves-Santos et al., 1999). After inoculation, the plants were either transferred to pots and further incubated in a greenhouse for regular pathogenicity tests, or to 50 ml Falcon tubes filled with PGM (Plant Growth Medium) solution and covered with foil. These hydroponic cultures were incubated in an environment-controlled chamber at 25°C and relative humidity of 60–80% with a 16 h photoperiod fluorescent light. All the strains and transformants were simultaneously tested in each inoculation experiment to reduce variability in the plant response due to environmental conditions. Pathogenicity tests were repeated three times and 12 replica plants were included per treatment.

External symptoms of disease in plants grown in Falcon tubes could not be correlated with those previously recorded using the CIAT scale in plants grown in pots, as growth of the plants under the hydroponic conditions used was slower. However, distinctions could be made between plants inoculated with the HV or the WV virulent strains, both in the number of chlorotic leaves and the degree of necrosis in the vascular system. A new scale was designed according to the degree of external (chlorotic leaves) and internal (necrotic lesions) symptoms observed. 0: healthy plant with no symptoms of chlorosis or necrosis; 1: plant with no chlorotic leaves but light necrotic lesions; 2: plant with some clorotic leaves and necrotic lesions in the crown region and the xylem system; 3: plant with most of the leaves either chlorotic or dead, extensive necrosis of the crown region and necrosis of the vascular system; 4: dead plant.

### Microscopic Examination by Means of Confocal Laser Microscopy

The plants inoculated with FOP-SP1 or FOP-SP4 and the controls treated with water were maintained in hydroponic cultures and examined each day for a period of 21 days after infection. For the examination of histological structure plant sections were fixed and embedded in paraffin as described (Jiménez et al., 2007), and then stained with toluidine blue 0.1% in potassium phosphate buffer 0.1 M pH 6.8. For CLSM analysis the plants were taken from the Falcon tubes and the roots washed in sterile distilled water. Plant sections or longitudinal samples 1 mm in thickness were manually sliced with a razor blade from the following tissues: root hairs, tap root zones (apex, intermediate, upper), crown and hypocotyl. Plant tissues were stained with a drop of propidium iodide (SIGMA) at a concentration of 10 mg/ml for 15 min and the excess propidium iodide was removed by washing with sterile distilled water. Then, samples were placed on microscope slides, submerged in glycerol 50% (v/v) and gently squashed with a cover glass. Microscopic analysis was performed using a laser scanning spectral confocal microscope (TCS2-SP2, Leica Microsystems, Germany). Excitation was provided by an argon laser (488 nm). Fluorescence of GFP was detected at 495–520 nm and plant tissue fluorescence (either propidium iodide stained and autofluorescence) at 595–680 nm.

The quantification of xylem vessel colonization was performed with the help of ImageJ. Data were obtained from at least six images per plant section showing growing hyphae of the HV strain or the WV strain. Parenchyma and xylem vessels were compared and GFP cell fluorescence was used to estimate the ratio of fungal colonization of similar areas in both tissues.

### Nucleic Acid Manipulations

Genomic DNA was extracted from *F. oxysporum* mycelium according to the procedures previously described (Alves-Santos et al., 1999, 2002b; Ramos et al., 2007). RNA was extracted from *P. vulgaris* plants at different times after inoculation with *F. oxysporum* strains. Roots and hypocotyls were cut, immediately frozen at -80°C and ground in a mortar with pestle under liquid nitrogen. Total RNA was extracted using TRIzol reagent (Invitrogen, Carlsbad, CA, USA), according to the manufacturer’s instructions. RNA was treated with DNAase Turbo DNA-free (Ambion Inc., Austin, TX, USA), tested for integrity by running samples in agarose gels and quantified using a Nanodrop Spectrophotometer (Thermo Scientific, Waltham, MA, USA). Southern blots were carried out as described previously (Ramos et al., 2007). DNA probes were labeled with digoxigenin-dUTP (Roche Diagnostics) by the PCR method using the Biotools DNA polymerase (Biotools SA, Spain) and plasmid pFR-HU-GFP DNA as template. Prehybridization, hybridization, washings, and detection were performed using a chemiluminescent detection procedure using CDP-Star (Roche Diagnostics) according to the manufacturer’s recommendations.

### Real-Time Quantitative Analysis of Gene Expression

Samples of plants inoculated with *F. oxysporum* were collected and frozen at -80°C. Total RNA was extracted as described before. cDNA was synthesized from 1 μg of total RNA in 20 μl (total volume) reactions using the PrimeScript^TM^ RT reagent kit (Takara Biosystems), and oligo (dT) as a primer, according to the manufacturer’s procedure.

The primer pairs used in RT-qPCR assays are shown in **Table 1**. Primer pairs used to analyze the expression of *F. oxysporum* genes were designed according to alignments performed using all the *F. oxysporum* genome sequences available at the Broad Institute database. They were verified in PCR experiments using genomic DNA from FOP-S1 and FOP-SP4 as template. All of them were able to amplify DNA fragments of the expected size in both strains, except the fragment corresponding to *FTF1*, as this gene is specific of HV strains. The amplicons were sequenced to verify their identities. Primer pairs used to analyze the expression of plant genes were designed on the basis of the *Glycine max* sequences. The amplicons obtained using common bean DNA as template were sequenced to confirm that they were homologues of the *G. max* genes. Before using a pair of primers in an expression analysis, its PCR amplification efficiency was calculated using six serial dilutions of cDNA as template. All the primer pairs tested showed efficiencies greater than 97%. qPCR was carried out in 10 μl reaction mixtures containing 1X KAPA SYBR Green qPCR Master Mix (Kapa Biosystems), 500 nM forward and reverse primers and 1 μl of the reverse transcription reaction. Amplifications were performed in a StepOnePlus^TM^ Real-Time PCR system (Applied Biosystems, Foster City, CA, USA) using the thermal profile recommended by the manufacturer (40 cycles of 95°C for 3 s and 60°C for 30 s). The presence of only one specific peak was checked in the melting curve (dissociation curve), which was run at 0.5°C increments every 5 s within a range of 55–95°C. The qPCR product size was checked by electrophoresis in 2.5% (w/v) agarose gels that were stained with ethidium bromide and visualized under UV light.

**Table 1:** Oligonucleotides used in this study.

Oligonucleotides	Ace. No.^∗^	Oligonucleotides sequence	Amplicon size (bp)
B310User	DQ280313^a^	GGGTTTAAUCAGCCATTCAT GGATGACATAACGAATTTC	670
5FTF1RBamHI		GGATCCGGCTCTGCCAGAGACAAAGTTAC	
5GFPFBamHI	gGFP^+^	GGATCCATGGTGAGCAAGGGCGAGGAG	782
GFPUserR		GGACTTAAUCCAGATTCGTCAAGCTGTTTG	
EBR1-F	FOXG_05408^b^	ACCACCGACAGCAACAGCAGC	155
EBR1-R		GTTGGGTCGGCGTTGATCCTC	
FNR1-F	DQ387858^a^	CCAGCAGTTCATGTATGGTGGCG	136
FNR1-R		CACCTGAGAGGGGTCGATATGCC	
FOW2-F	AB266616^a^	CAAATCCTTCGCCCTCACATCTG	130
FOW2-R		GATCCTGGTGTCAAAGCCGAGG	
FTF1-F	DQ280313^a^	TGTGGTGGCCAGGATATGATG	110
FTF1-R		TGCATGCCTGCCTTGACAT	
FTF2-F	FOXG_09390^b^	ATGCTCACACCCCCACATTCT	108
FTF2-R		ATCCCCCAAAGACAAGCTGAC	
PacC-F	AY125958^a^	CATGGCAACCTCTCCGTTCCC	132
PacC-R		GGGCAGGTAGTATTGCTGAGCCG	
RHR1-F	FOXG_05541^b^	CCATGCTGAGATTCTCCACGGC	175
RHR1-R		CATGACAGGATCGGTTTGGGGTG	
RHR2-F	FOXG_09999^b^	CAAGTGGATGGGAGACCTACGCC	158
RHR2-R		TGAGCCTGCATTGGCGATTGG	
SGE1-F	FOXG_10510^b^	CAGCCGTATCCTTGGCAACTA	101
SGE1-R		TGGTTGACTTGCCGTTCCTT	
SIX1-F	AJ608702^a^	GAGCCGCCTCAATCGCCTG	194
SIX1-R		GCCCAAGTTGCGCGATATGTG	
SIX6-F	ACY39286.1^a^	GCTTTTGCGTGGCGAACCC	102
SIX6-R		TTTTCCCGTTGCTGAGATTGCG	
PR1-F	Phvul.003G109100^c^	CAGGCACTACACTCAGGTTGTTTGGA	111
PR1-R		TTGCCAGGAGGAGCATAGTTGCAA	
PR5-F	Phvul.001G016700^c^	CGGAAAATGTGTCACCGGAGACTG	148
PR5-R		ATTGTAACCGTCCACCAGGCTCAC	
ERF1-F	Phvul.007G127800^c^	CCTGTTGTGGCTCTGAAGAGGAAAC	124
ERF1-R		CAGGACCAAGGTCTTCAAACACGAC	
ERF2-F	XM_003549886^d^	GGGAAAGTTCGCGGCGGAG	164
ERF2-R		CGGAGTTAACCCTCAACGGAAAATTC	
EF1alpha-F	FOXG_03515^b^	CATCGGCCACGTCGACTCT	144
EF1alpha-R		AGAACCCAGGCGTACTTGAA	
Actin-F	Phvul.011G064500^c^	GAAGTTCTCTTCCAACCATCC	175
Actin-R		TTTCCTTGCTCATTCTGTCCG

*^∗^GeneBank numbers ^(a)^ or loci numbers of the F. oxysporum genome ^(b)^ sequence (http://www.broadinstitute.org/annotation/genome/fusarium_group/MultiHome.html), the P. vulgaris genome ^(c)^ (http://www.phytozome.net/commonbean.php) or the Glycine max genome ^(d)^ (http://www.phytozome.net/soybean.php) are provided, except ^+^plasmid gGFP.*

Two or three sets of samples from different infection assays were used (biological replicates). Two independent cDNA preparations per biological replicate were obtained, and three replicas of each cDNA were analyzed. The endogenous reference genes used were the *F. oxysporum* *EF1a* encoding a translation elongation factor and the common bean actin gene. The expression of both genes was found to remain constant under all the conditions used in the present study (data not shown). Relative expression levels were calculated using the comparative cycle threshold (Ct) method (Bustin, 2000; Pfaﬄ et al., 2002), using the formula RQ (relative quantity of transcript) = 2^-ΔΔCt^, where ΔCt = Ct_specific gene_ – Ct_reference gene_ of any given sample, and ΔΔCt = ΔCt (as previously calculated) – ΔCt of 1 dpi FOP-SP4 inoculated samples, when specific fungal genes where analyzed, or ΔCt of mock inoculated plants, when plant genes were analyzed. All Ct calculations, integration of the amplification efficiency data and statistical analyses were performed using StepOne^TM^ Software V2.2.2.

### Fungal Biomass Quantification

The relative fungal DNA amount was quantified by means of qPCR. Six infected plants were randomly selected and collected for each condition assayed, and DNA obtained as previously described. For the detection and quantification of fungal DNA primers that generate fragments of the *EF1*α and *FTF2* (Fusarium Transcription Factor 2) genes were used (**Table 1**). Both genes are found as single-copy sequences in the genome of *F. oxysporum*. A fragment of the actin gene from common bean, generated by using primers Actin-F and Actin-R (**Table 1**), was used as an endogenous gene to normalize differences in DNA template amounts. The qPCR reaction components and cycling conditions were as above described, except that 1 ng of DNA was used as template.

Fungal biomass in inoculated samples was estimated as the relative quantity of FOP-SP1 or FOP-SP4 DNA normalized to an endogenous control of *P. vulgaris* DNA. Standard curves for *F. oxysporum* and *P. vulgaris* were constructed based on the relationship of Ct values and known host and pathogen DNA concentrations. Genomic DNA isolated from fungal mycelia grown in culture and samples of roots, root crowns, and hypocotyls of non-inoculated plants were used to create the standard curves. Each sample was evaluated in triplicate and the assay was repeated two times for each of the three biological replicates. The analyses were performed using StepOne^TM^ Software V2.2.2.

## Results

### Cytological Characterization of Colonization by Weakly Virulent and Highly Virulent Strains of *F. oxysporum* f. sp. *phaseoli*

Green fluorescent protein transformants of *F. oxysporum* f. sp. *phaseoli* were generated by transformation of strains FOP-SP1 and FOP-SP4 with plasmid pRF-HU-GFP. Both strains are wild types isolated in El Barco de Ávila (Spain) that share IGS-A haplotype, mating type (MAT1-2) and race (6). However, FOP-SP1 is a HV strain that belongs to VCG 167, while FOP-SP4 belongs to VCG 166 and behaves as WV when inoculated on common bean plants (Alves-Santos et al., 1999, 2002a). Several GFP expressing transformants were obtained for each strain and examined for mycelial growth, both in solid and liquid culture, sporulation and virulence. No significant differences could be observed in the rates of growth in PDA medium and rates of sporulation in PDB medium between the GFP transformants and their respective wild type strains. Infection assays were also performed to compare GFP expressing strains with the wild type strains by inoculation of cultivar Blanca Riñón, but no significant differences (*P* > 0.9 according to “Student’s” *t*-test) could be observed when the mean disease symptoms or the disease progression rate (García-Sánchez et al., 2010) were assessed (data not shown).

**FIGURE 1:**
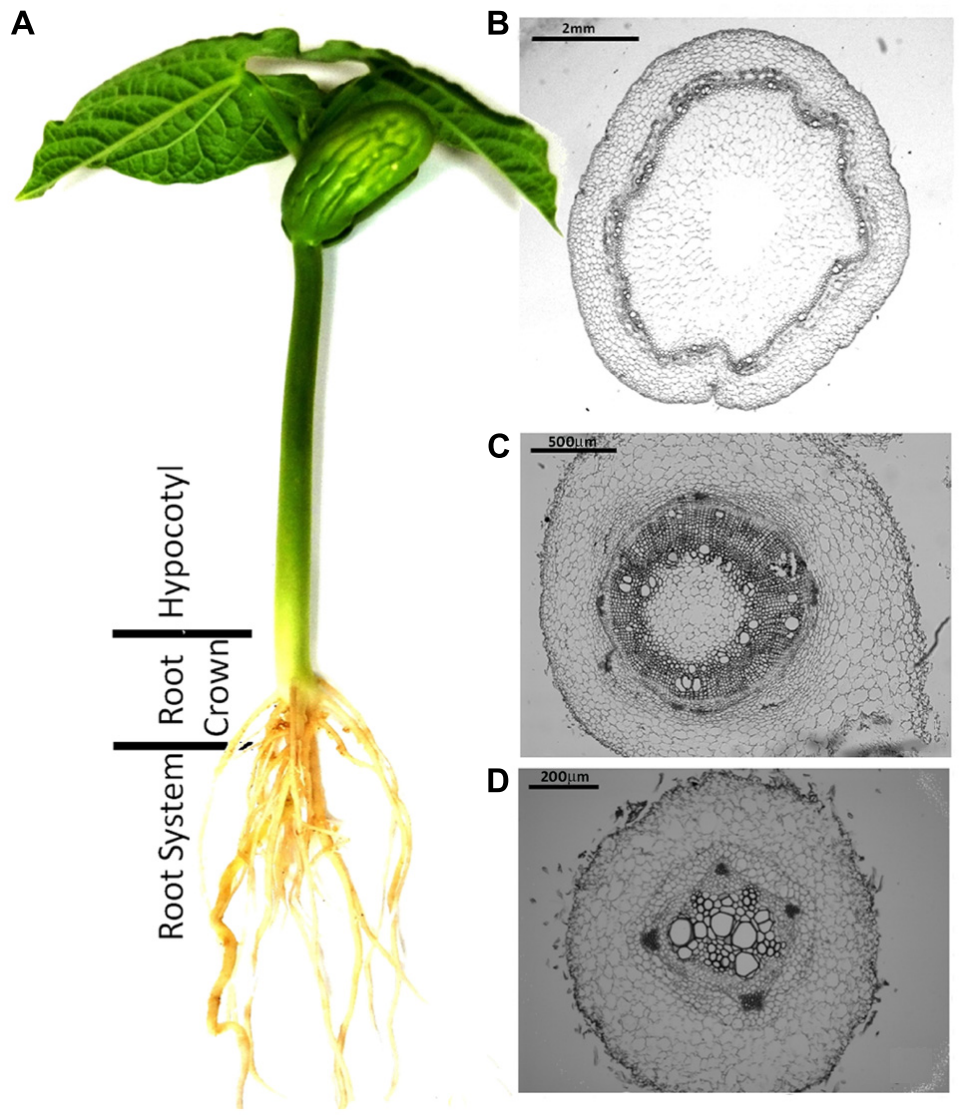
**Common bean plant (*Phaseolus vulgaris* L.) and histological structure of the plant regions analyzed in the present study.** **(A)** Common bean plant grown in hydroponic culture. **(B–D)** Cross sections of the three plant regions analyzed in the present study showing the definitive architecture of the xylem and phloem vessels in the hypocotyl **(B)**, the primordial rings of xylem and phloem vessels in the root crown **(C)** and the undifferentiated central cylinder in the tap root **(D)**. Plant sections were fixed, embedded in paraffin and stained with toluidine blue for microscopic examination.

**FIGURE 2:**
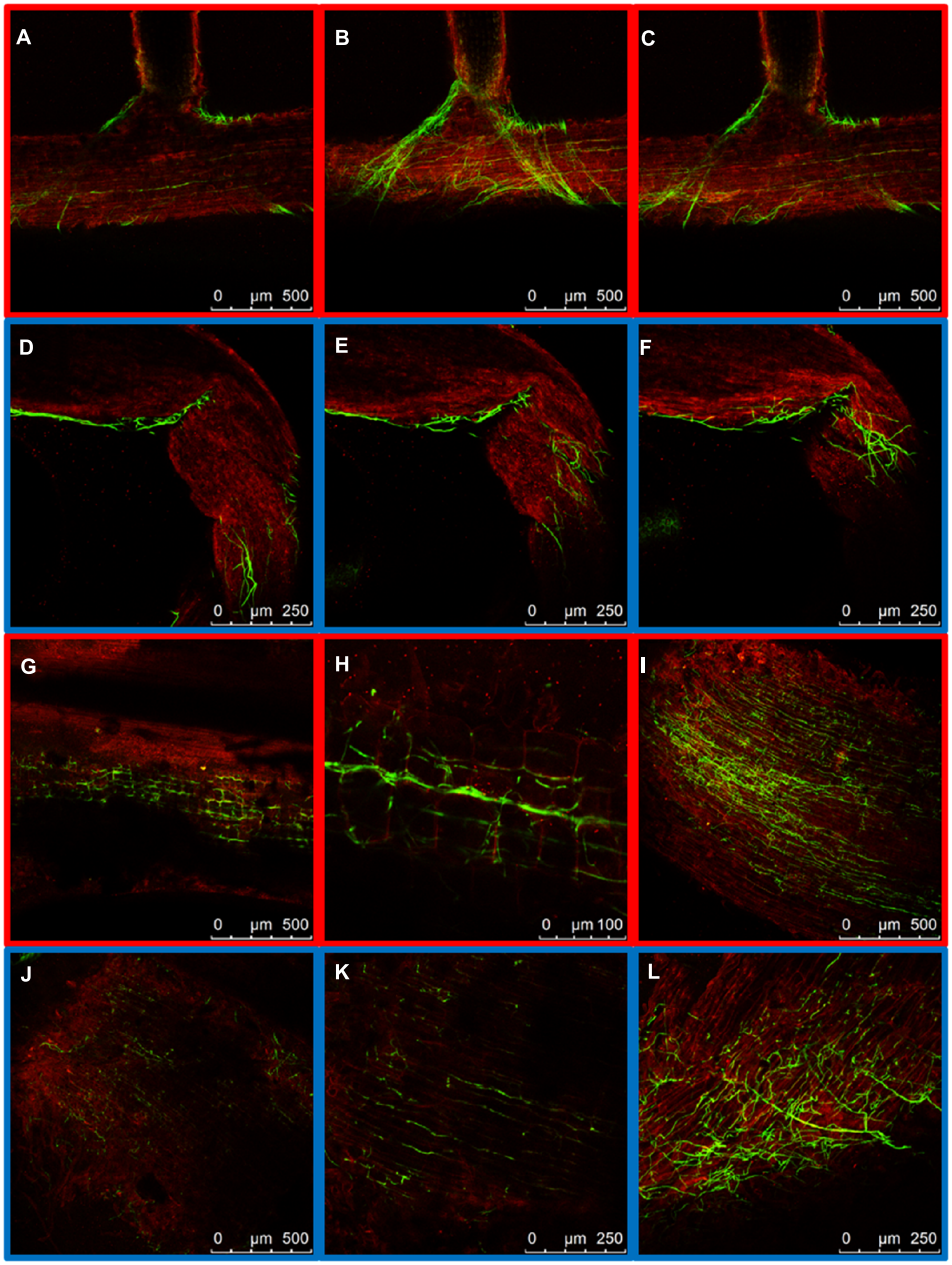
**External colonization of the root system by highly virulent (HV; FOP-SP1) and weakly virulent (WV; FOP-SP4)** *Fusarium oxysporum* f. sp. *phaseoli* strains. Colonization of the taproot and lateral roots of plants inoculated with the HV strain FOP-SP1 (red framed images) and the WV strain FOP-SP4 (blue framed images) was visualized by confocal laser scanning microscopy 1–3 days post inoculation (dpi). **(A–C)** FOP-SP1 mycelium growing in the junction of a lateral root with the taproot. **(D–F)** FOP-SP4 mycelium growing in an interstitial zone of a lateral root. Intermediate root zones showing epidermis colonization by FOP-SP1 hyphae **(G–I)** and FOP-SP4 hyphae **(J–L)**.

To compare the patterns of common bean colonization by WV and HV strains three different regions of inoculated common bean plants were chosen for confocal microscopy based on the different architecture of their vascular system: the tap root, the root crown and the hypocotyl (**Figure 1**). The initial stages of root colonization were similar in plants inoculated with WV (FOP-SP4) and HV (FOP-SP1) strains. Following root dip inoculation, a dense web of hyphae grew over the surface of the taproot and the lateral roots (**Figures 2G-L**). Both strains showed a marked preference for the colonization of interstitial regions, especially the junctions of lateral roots with the tap root (**Figures 2A-F**) which appear to serve as preferred penetration areas. Specialized penetration structures could not be observed for any of the strains.

**FIGURE 3:**
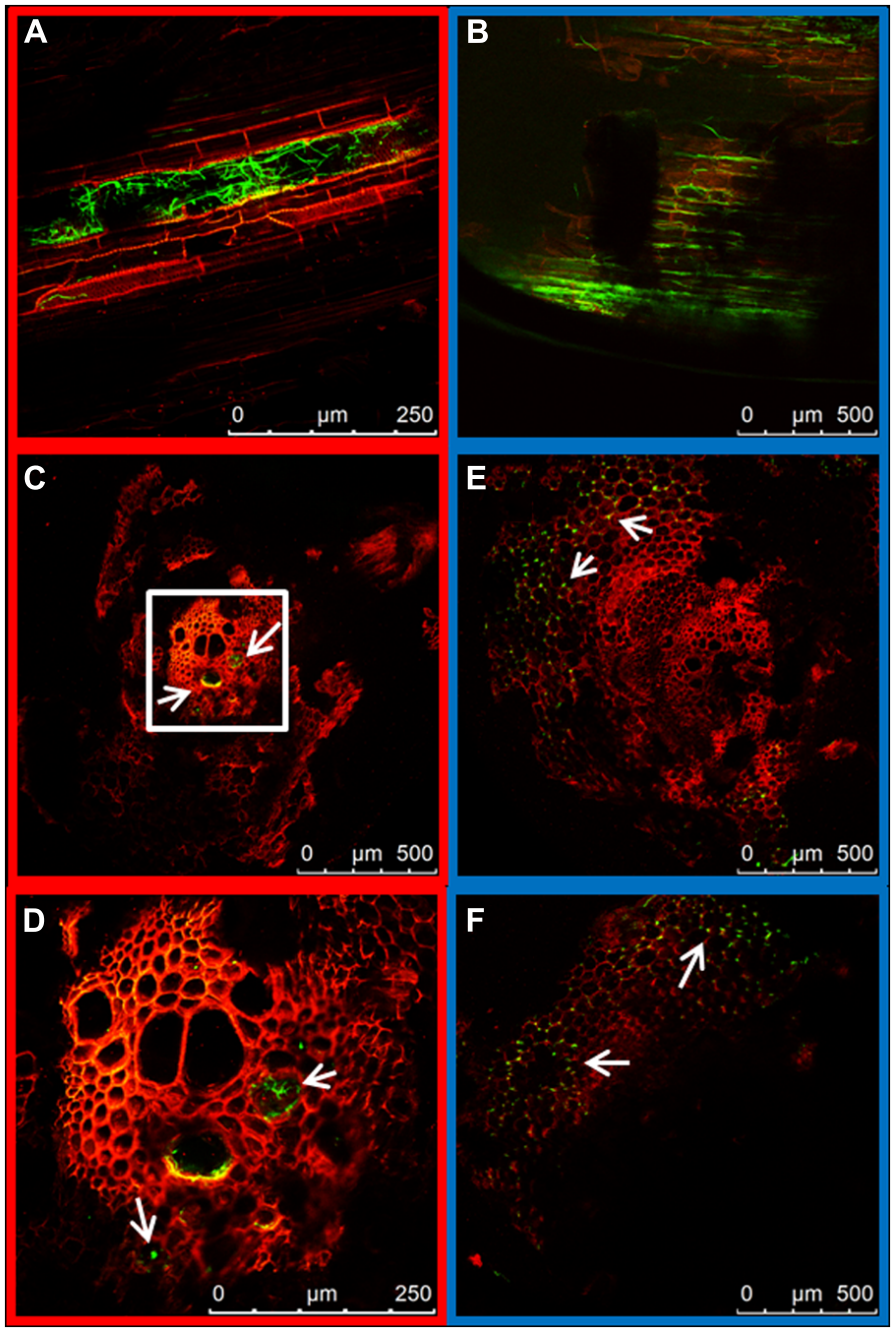
**Internal colonization of the root system by HV (FOP-SP1) and WV (FOP-SP4) *F. oxysporum* f. sp. *phaseoli* strains.** Mycelial growth of FOP-SP1 and FOP-SP4 inside the taproot was visualized in longitudinal sections **(A,B)** and cross sections **(C–F)** of infected plants 1–3 dpi. The arrows indicate hyphae of FOP-SP1 growing inside the vessels of the central cylinder of the taproot (red framed images) and hyphae of FOP-SP4 colonizing the cortex of the taproot (blue framed images).

At 7 days post inoculation (dpi) the taproots were almost completely colonized by both strains. However, a clear distinction in the main distribution of the mycelium could be observed. The WV strain showed extensive intercellular growth in the root cortex, mainly along the longitudinal axis of the root (**Figure 3B**), although some hyphae could also be seen inside the xylem vessels of the central root cylinder. In contrast, the hyphae of the HV strain quickly reached the central root xylem where they spread along the xylem vessels (**Figure 3A**). Comparison of cross sections of the taproot clearly demonstrates how the WV strain behaves mainly as a root cortex colonizer (**Figures 3E,F**) while the HV strains behaves as a fast vascular colonizer (**Figures 3C,D**).

**FIGURE 4:**
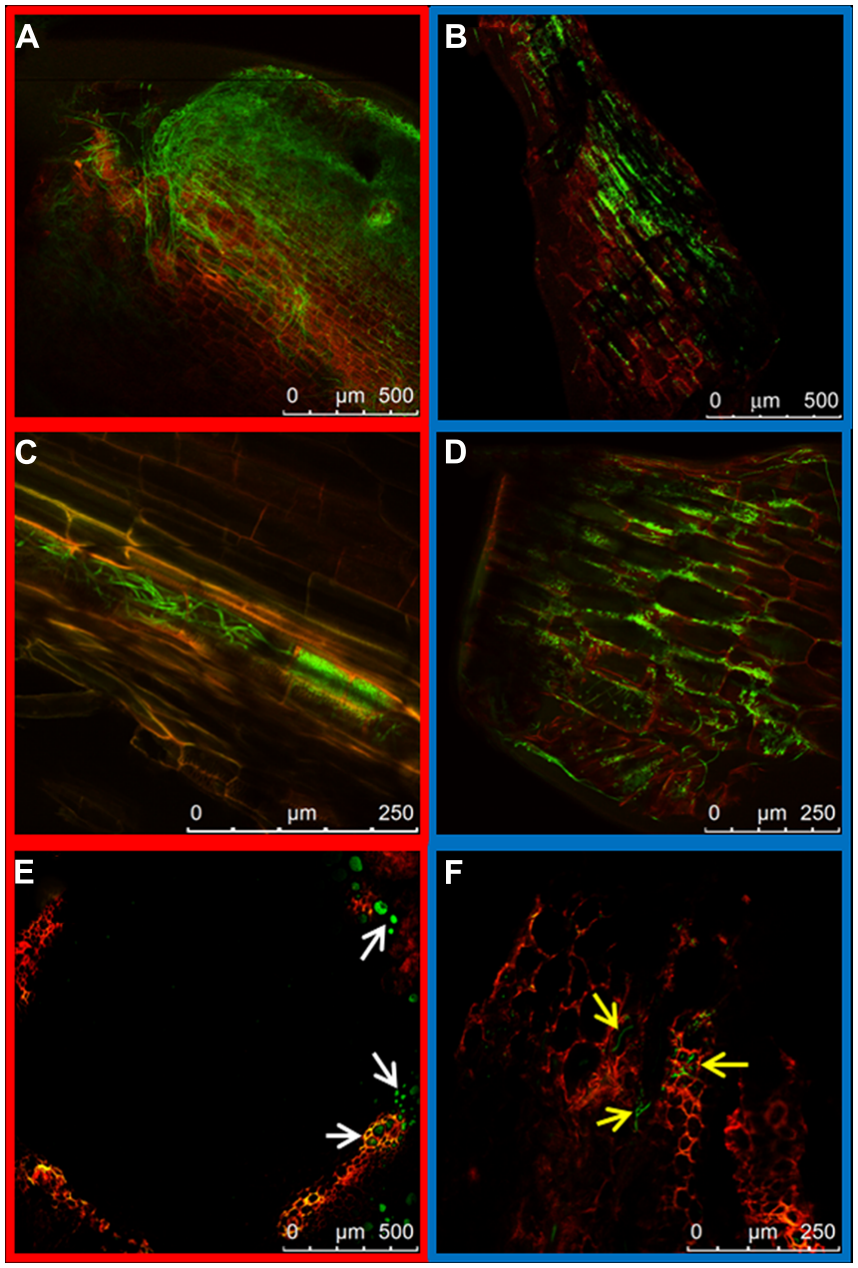
**Colonization of the root crown by HV (FOP-SP1) and WV (FOP-SP4) *F. oxysporum* f. sp. *phaseoli* strains.** The growth of mycelium of FOP-SP1 and FOP-SP4 in the root crown region was visualized in longitudinal sections **(A–D)** and cross sections **(E,F)** of plants at 5–7 dpi. The hyphae of FOP-SP1 heavily colonized the cortex **(A)** and the xylem vessels (**C** and white arrows in **E**) of the root crown. The growth of hyphae of FOP-SP4 in the root crown predominantly occurred along the interstitial spaces of cortical cells **(B,D)** although some hyphae also colonized the xylem vessels (yellow arrows in **F**).

Fungal mycelia could also be seen in the root crown at 7 dpi. Two differences could be observed between the two strains. First, root crowns of plants inoculated with the HV strain had more green fluorescent hyphae than those inoculated with the WV strain (**Figures 4A,B** respectively). Second, the asymmetrical distribution of hyphae in xylem vessels and the intercellular spaces of cortex, observed in the taproot, was maintained. The colonization of the vascular system by the HV strain was preferentially observed in adjacent vessels, indicating that hyphae can penetrate the vessels (**Figures 4C,E**). Mycelium of the WV strain was mostly detected around the cells of the cortex, although some hyphae could also be seen growing inside the xylem vessels (**Figures 4D,F**). Plants inoculated with the HV strain were rated 1 and those inoculated with the WV strain were rated 0.

**FIGURE 5:**
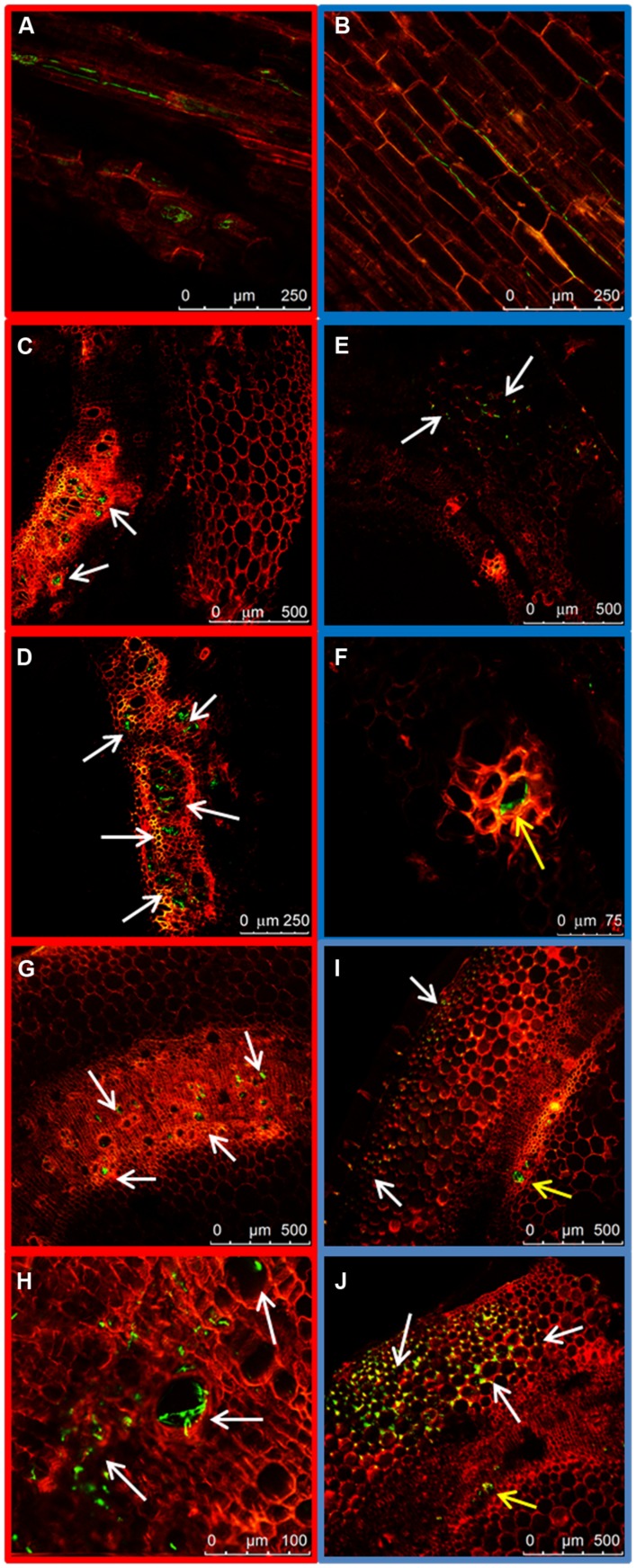
**Colonization of the hypocotyl by HV (FOP-SP1) and WV (FOP-SP4) *F. oxysporum* f. sp. *phaseoli* strains.** The growth of mycelium of FOP-SP1 (red framed images) and FOP-SP4 (blue framed images) was visualized in longitudinal sections **(A,B)** and cross sections **(C–J)**. Hyphae of FOP-SP1 were detected growing inside the xylem vessels almost exclusively (arrows) at 10 dpi **(A)**, 14 dpi **(C,D),** and 21 dpi **(G,H)**. Hyphae of FOP-SP4 were mainly detected growing along the cells of cortex and parenchyma (white arrows), at 10 dpi **(B)**, 14 dpi **(E,F)** and 21 dpi **(I,J)**, although some hyphae were also seen inside the xylem vessels (yellow arrows).

Early observations of plant hypocotyls (10 dpi) showed hyphae of the HV strain colonizing the inside of the xylem vessels (**Figure 5A**) while the hyphae of the WV strain were preferentially seen along the walls of parenchymal cells in longitudinal sections (**Figure 5B**). Inoculated plants were rated 2 and 1, respectively. This observation and the difference in the ratings were confirmed in cross sections at 14 dpi (**Figures 5C,E**) when disease rates reached 3 for plants inoculated with the HV strain and 2 for plants inoculated with the WV strain. Although xylem vessels containing growing hyphae were observed for both strains (**Figures 5D,F**), the incidence (proportion of colonized vessels) was dramatically different, reaching 81.06% (±8.88) for the HV strain and 11.39% (±7.03) for the WV strain. Cross sections of plants 21 dpi showed massive amounts of hyphae of the HV strain growing almost exclusively inside the xylem vessels (**Figures 5G,H**), while most of the hyphae of the WV strain were around the parenchymal cells (**Figures 5I,J**). At this time, plants inoculated with the HV strain were almost dead and showed complete necrosis of the crown region and extensive necrosis of the xylem system of the hypocotyl (disease rate 4). Plants inoculated with the WV strain had some chlorosis, necrotic crown and some symptoms of necrosis in the xylem system of the hypocotyl (disease rate 3).

### Quantification of Fungal Growth

In order to determine whether the patterns of colonization showed by both strains were correlated to differences in fungal biomass, the growth of both strains of the pathogen inside the plant was evaluated by means of a qPCR assay. *F. oxysporum* DNA relative to that of common bean was measured by assaying the *F. oxysporum EF1*α and *FTF2* genes of FOP-SP1 and FOP-SP4 strains, and the *P. vulgaris* *actin* gene.

**FIGURE 6:**
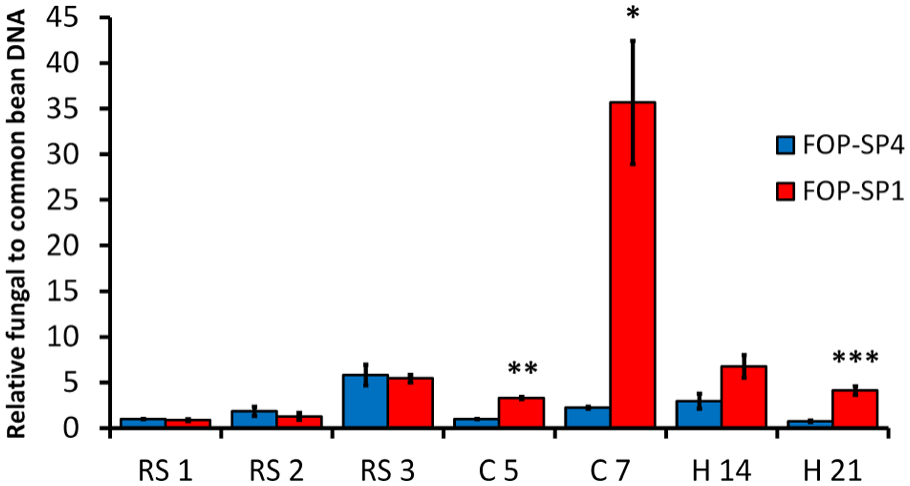
**Quantification of fungal biomass in *P. vulgaris* plants colonized by HV and WV strains of *F. oxysporum* f. sp. *phaseoli*.** FOP-SP4 DNA (blue bars) or FOP-SP1 DNA (red bars) relative to that of common bean was measured by assaying the fungal *FTF2* gene and the plant *actin* gene by RT-qPCR using DNA extracted from the root system at 1, 2, and 3 dpi (RS1, RS2, and RS3), root crown at 5 and 7 dpi (C5 and C7) and hypocotyl at 14 and 21 dpi (H14 and H21). All measurements were referred to the value obtained for FOP-SP4 colonization at RS1 (arbitrary value of 1.0). Significative differences were observed for C5 (**p < 0.01), C7 (^∗^*p* < 0.05), and H21 (^∗∗∗^*p* < 0.001).

The accumulation of fungal biomass in the root system was similar in plants inoculated either with the HV strain or the WV strain, and reached a maximum at 3 dpi (**Figure 6**). In contrast, The accumulation of mycelium both in the root crown region and the hypocotyl was higher in plants inoculated with the HV strain than in plants inoculated with the WV strain: 16 times more in root crown at 7 dpi and more than five times in hypocotyl at 21 dpi (**Figure 6**).

Taking together the confocal microscopy observations and the qPCR fungal biomass quantification results, two main differences emerge in the colonization pattern of HV and WV strains. First, the HV strain is a specialized vascular colonizer, while the WV strain, although also able to colonize the vascular system, is better at colonizing cortex and parenchyma. Second, the HV strain accumulates a significantly higher amount of mycelium in plant aerial regions than the WV strain.

### Expression Analysis of Selected Fungal Virulence Genes

**FIGURE 7:**
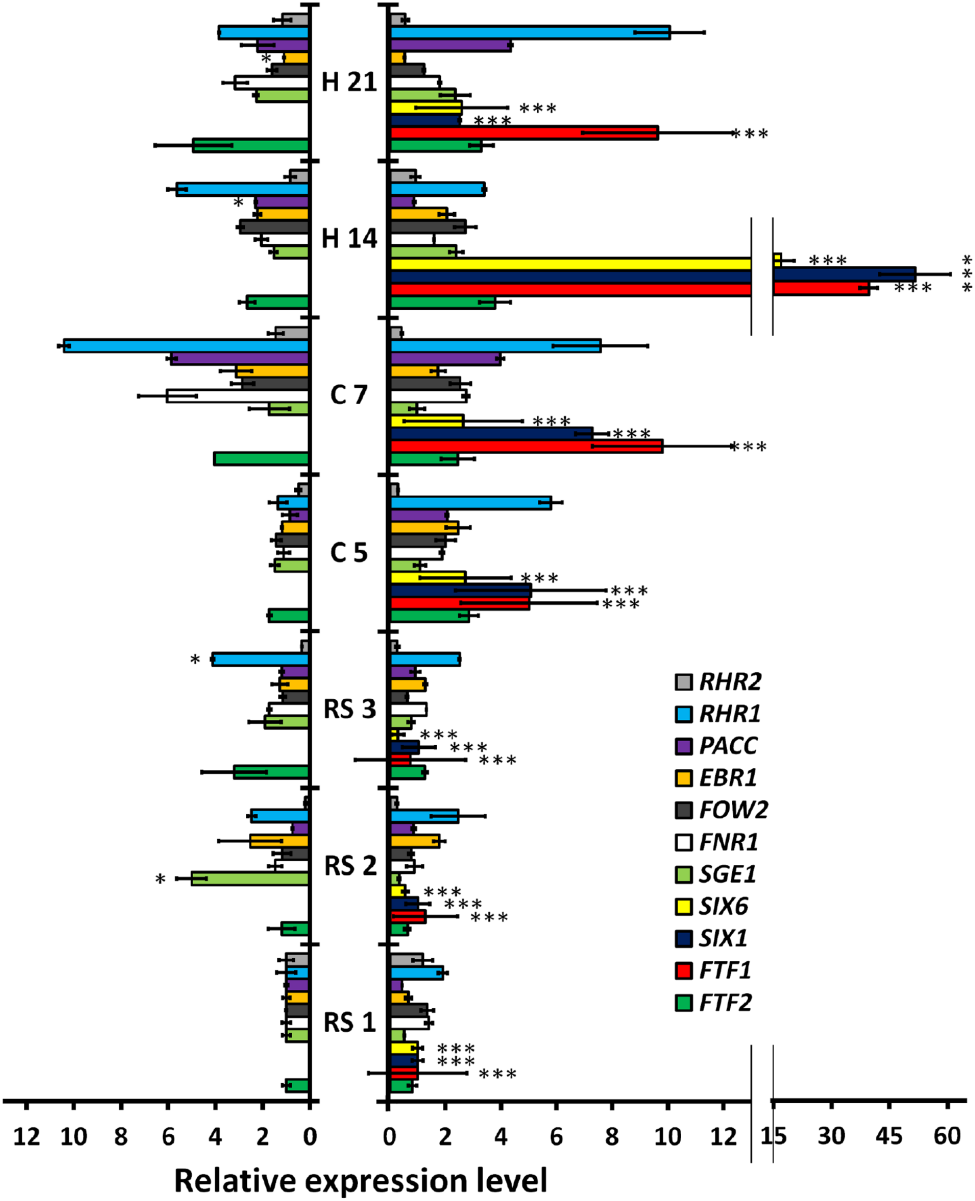
**Quantitative reverse transcription polymerase chain reaction (RT-qPCR) analysis of time-course expression of eleven fungal genes in *P. vulgaris* plants colonized by HV and WV strains of *F. oxysporum* f. sp. *phaseoli*.** The plant regions assayed and the time intervals after inoculation are indicated in the center of the figure: root system at 1, 2, and 3 dpi (RS1, RS2, ans RS3, respectively); root crown at 5 and 7 dpi (C5 and C7, respectively); and hypocotyl at 14 and 21 dpi (H14 and H21, respectively). The bars on the left show the results obtained in common bean plants colonized by the WV strain (FOP-SP4). The bars on the right show the corresponding results for plants colonized by the HV strain (FOP-SP1). The expression ratios were normalized by using the fungal *EF1*α gene as an endogenous control. An arbitrary value of 1.0 was denoted for the transcript level of each gene in plants colonized by the WV strain at RS1, except for the FTF1, SIX1, and SIX6 transcripts whose 1.0 values correspond to the transcript level of each gene in plants colonized by the HV strain at RS1. The results shown are the averages and their respective standard deviations obtained in three independent biological experiments. The expression level value differences between each pair of samples (the expression of the same gene in plants colonized by the HV strain or the WV strain) were tested using the *t*-test and indicated by (*p < 0.05) and (***p < 0.001).

In order to understand the genetic basis of the differential colonization patterns displayed by HV and WV strains, we analyzed the *in planta* changes of expression of a selected group of genes already shown to be involved in virulence or suspected to be virulence factors (**Table 2**). RNA samples were purified from root (1, 2 and 3 dpi), crown (5 and 7 dpi) and hypocotyl (14 and 21 dpi) and used in RT-qPCR experiments to measure the expression of each gene analyzed (**Figure 7**). The most relevant changes of gene expression in root samples were the upregulation of *RHR1* and *EBR1* in both strains and the specific upregulation of *SGE1* (fivefold increase) and *FTF2* (threefold increase) in the WV strain. A drastic change in gene expression pattern related to the virulence of each strain was observed in crown at 5 dpi. With the exception of *SGE1* and *RHR2* all the genes analyzed showed upregulation (twofold or more) during the infection with the HV strain, reaching highest levels for *FTF1*, *SIX1,* and *RHR1* (more than fivefold increase). Two days later (7 dpi) the same plant region showed significant upregulation of *FNR1*, *PacC*, *FTF2* and *RHR1* in the WV strain. Relatively high expression patterns (threefold or more) in hypocotyl colonized by the WV strain remained for *RHR1* (14 and 21 dpi), *PacC* and *FTF2* (21 dpi), and in the case of hypocotyl colonized by the HV strain, for *PacC* (21 dpi), *FTF2* (14 and 21 dpi) and most significantly for *FTF1* (14 dpi) and *SIX1* and *RHR1* (14 and 21 dpi).

**Table 2:** Fungal genes analyzed during colonization of *Phaseolus vulgaris* plants by weakly and highly virulent strains of *F. oxysporum*.

Gene	Ace. No^∗^	Product^+^	Effect of gene inactivation	Reference
*EBR1*	FOXG_05408 ^b^	TF	Reduced virulence; impaired growth; reduced biocontrol capacity	Zhao et al. (2011)
*FNR1*	DQ387858^a^	TF	Reduced virulence; reduced ability to use secondary nitrogen sources	Divon et al. (2006)
*F0W2*	AB266616^a^	TF	Non-pathogenic; impaired in invasive growth	Imazaki et al. (2007)
*FTF1*	DQ280313^a^	TF	Reduced virulence	Ramos et al. (2007), Niño-Sánchez (unpublished)
*FTF2*	FOXG_09390^b^	TF	Unknown; homologue of *FTF1*	Ramos (2005)
*PacC*	AY125958^a^	TF	Increased virulence and expression of acid-expressed genes	Caracuel et al. (2003)
*RHR1*	FOXG_05541^b^	TF	Impaired to use L-rhamnose as carbon source	Pardo and Orejas (2014)
*RHR2*	FOXG_09999 ^b^	TF	Unknown	Orejas (pers. comm)
*SGE1*	FOXG_10510^b^	TF	Non-pathogenic; reduced sporulation	Michielse and Rep (2009)
*SIX1*	AJ608702^a^	SSP	Reduced virulence (more pronounced in older plants)	Rep et al. (2004)
*SIX6*	ACY39286.1^a^	SSP	Reduced virulence	Gawehns et al. (2014)

*^∗^GenBank accesion numbers ^(a)^ or loci numbers of the F. oxysporum genome sequence ^(b)^(http://www.broadinstitute.org/annotation/genome/fusarium_group/MultiHome.html) are provided. +TF, transcription factor; SSP, small secreted protein.*

The cellular basis of the host-pathogen interaction in vascular wilt diseases was first described using transmission electron-microscopy (Bishop and Cooper, 1983). The use of laser scanning spectral confocal microscopy (CLSM) has improved our knowledge of the details concerning infection in multiple hosts and different races of the pathogen. The majority of studies show that invasion of the host by *F. oxysporum* strains expressing the green fluorescent protein (GFP) begins with the development of a hyphal network around root hairs and the upper part of taproot, followed by penetration and colonization of the root epidermis. Once the hyphae are inside the roots the development of disease symptoms and degree of colonization in resistant and susceptible cultivars show differences in several hosts. *F. oxysporum* f. sp. *medicaginis* colonizes the cortex and central cylinder of roots of resistant and susceptible cultivars in similar ways. In *Medicago truncatula* inoculation of a resistant cultivar resulted in the delay of symptom development and reduction of the disease severity with respect to the inoculation of a susceptible cultivar, but no correlation could be found with the degree of colonization of the root parenchyma and central cylinder (Ramírez-Suero et al., 2010). In watermelon, the pathogen can colonize the lateral roots in compatible interactions, but it is unable to penetrate the taproot when infecting a resistant cultivar (Lü et al., 2013). A more complex interaction has been recently reported in the infection of chickpea with *F. oxysporum* f. sp. *ciceris*. In compatible interactions the pathogen grows intercellularly in the root cortex, reaches the xylem and then advances upward through the xylem of the stem (Jiménez-Fernández et al., 2013). All the incompatible interactions were asymptomatic, but a race 0 strain was able to colonize the xylem vessels of root and stem of the resistant cultivar JG-62, while in another resistant cultivar, WR-315, it was halted in the intercellular spaces of the root cortex failing to reach the xylem. However, a race 5 strain progressed up to the hypocotyl in the same resistant cultivar WR-315 (Jiménez-Fernández et al., 2013). Therefore, colonization of xylem vessels does not guarantee a compatible interaction, at least in chickpea.

All the examples described above are based on the analysis of host-specificity (formae speciales) and cultivar specificity (races). Much less attention has been paid to interactions involving more virulent or less aggressive strains, even though virulence is often the only difference between a devastating and a tolerable disease. Previously, we characterized several groups of virulence in *F. oxysporum* f. sp. *phaseoli* and demonstrated that virulence is not correlated with race, because strains that belong to the same race may display different virulence on the same common bean cultivar (Alves-Santos et al., 2002a). However, virulence depends in part on the host, because equally virulent strains on *P. vulgaris* show differences in virulence on *P. coccineus* (de Vega-Bartol et al., 2011). We have also shown that some virulence factors are specific to highly virulent (HV) strains (Alves-Santos et al., 2002b) and specifically induced *in planta* (Ramos et al., 2007). In the only confocal microscopy study reported to date involving strains of *F. oxysporum* differing in virulence, we obtained evidence about the importance of the speed of xylem vessel colonization as a determinant of disease severity (García-Sánchez et al., 2010). This analysis left open some important questions related to the differences in colonization between HV and weakly virulent (WV) strains. For example, do HV strains develop a higher amount of fungal biomass inside the host than WV strains? What are the spatial milestones of colonization that differentiate both types of strains? Finally, if there is a differential expression of fungal virulence factors and plant defense factors, when does it happen?

In this work we addressed the above questions by determining the qualitative and quantitative differences on the spatial and temporal dynamics of colonization of a susceptible common bean cultivar infected by WV and HV strains of *F. oxysporum* f. sp. *phaseoli*, the gene expression patterns of several fungal virulence factors, and the host defense response involving expression of genes related to the salicylic acid (SA) defense induced response and the ethylene (ET)/jasmonic mediated response. Systemic acquired resistance (SAR) confers protection against a broad spectrum of microorganisms. SAR requires the signal molecule SA and is associated with accumulation of pathogenesis-related proteins (PRs; Durrant and Dong, 2004). Also, the cross-talk between ET and jasmonate (JA) signaling pathways determines the activation of defense responses against pathogens.

## Materials and Methods

### Fungal Strains and Culture Conditions

The *F. oxysporum* f. sp. *phaseoli* strains FOP-SP1 (HV) and FOP-SP4 (WV) used in this study have been described (Alves-Santos et al., 1999, 2002a). All strains were grown as previously described (Alves-Santos et al., 1999). Fungal cultures were established from frozen mycelia stored on 25% glycerol (v/v) at -80°C, and incubated at 25°C with continuous light for 1 week (solid media) or 5 days at 120–180 rpm (liquid cultures).

### Development of GFP-Expressing Strains of *F. oxysporum*

Plasmid pRF-HU-GFP was constructed to express GFP in *F. oxysporum* f. sp. *phaseoli*. A 3.1 Kb fragment containing the sGFP gene under the control of the glyceraldehyde 3-phosphate deshydrogenase promoter of *Aspergillus nidulans* was excised from plasmid gGFP (Maor et al., 1998; Sukno et al., 2008) by digestion with *Xba*I and *Cla*I. This 3.1 Kb *Xba*I-*Cla*I fragment was blunt-ended using the Klenow fragment of T4 DNA polymerase and ligated into the binary vector pRF-HU (Frandsen et al., 2008) digested with *Pac*I and also blunt-ended. Plasmid pRF-HU-PFTF1GFP was designed to express GFP under the control of the *FTF1* promoter (P*FTF1*). The pair of primers B310User-5FTF1RBamHI (**Table 1**) was used to amplify a 670 bp DNA fragment containing the promoter of gene *FTF1* using genomic DNA of strain FOP-SP1 as template. In parallel, primers 5GFPFBamHI and GFPUserR were used to amplify the coding region of sGFP using as template the plasmid gGFP. Both PCR amplification products were digested with *Bam*HI (as recognition sequences were included in one of the primers of each pair) and then ligated using T4 DNA ligase. 100 μl of the ligation reaction were used as template to amplify the cassette PFTF1::GFP. The primers used to this end (B310User and GFPUserR) included the sequences needed to ligate the PCR product to plasmid pRF-HU digested with *Pac*I and *Nt.Bbv*CI USER^TM^ enzimes (Frandsen et al., 2008). Both plasmids, pRF-HU-GFP and pRF-HU-PFTF1GFP, were used to genetically transform *F. oxysporum* f.sp. *phaseoli* by means of the *A. tumefaciens*-mediated transformation procedure as previously described (Mullins et al., 2001; Ramos et al., 2007).

Significant differences in the gene expression patterns between both strains were observed for *FTF1*, *SIX1* and SIX6. The difference is obvious for *FTF1*, as it is specific to the HV strains, but both *SIX1* and *SIX6* are present in the genome of the WV strain although no expression could be detected at any stage of the infection process. Also, it is worth to note the upregulation of *SGE1* in root tissues colonized by the WV strain at 2 dpi, and the upregulation of *RHR1* in root crown and hypocotyl colonized by the HV strain at 5 and 21 dpi, respectively.

### Expression Analysis of Selected Plant Defense Genes

**FIGURE 8:**
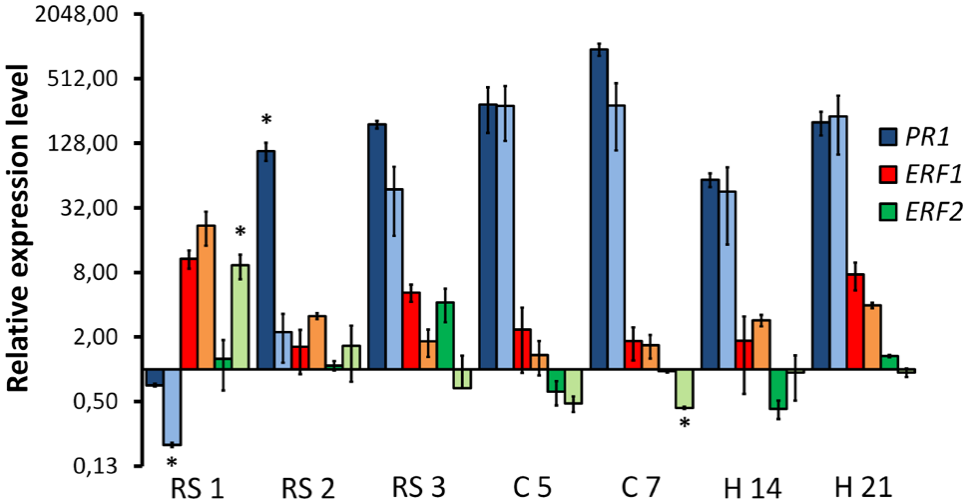
**Quantitative reverse transcription polymerase chain reaction analysis of time-course expression of common bean genes involved in the defense response in plants colonized by HV and WV strains of *F. oxysporum* f. sp. *phaseoli*.** The plant regions assayed and the time intervals after inoculation are indicated in the *X* axis: root system at 1, 2, and 3 dpi (RS1, RS2, and RS3, respectively); root crown at 5 and 7 dpi (C5 and C7, respectively); and hypocotyl at 14 and 21 dpi (H14 and H21, respectively). The relative expression measurements in the *Y* axis are indicated in a logarithmic scale. Dark bars for each color indicate expression of the corresponding gene in plants colonized by the WV strain, and light bars indicate expression of the corresponding gene in plants colonized by the HV strain. The expression ratios were normalized by using the common bean *actin* gene as endogenous control. The levels of expression for a pair of measurements (dark and light bars) were tested using the *t*-test and significative differences (*p* < 0.05) indicated by ^∗^.

We investigated the response of the pathogenesis related gene *PR1* and two ET responsive factors (*ERF1* and *ERF2*) in the plant tissues colonized either by the WV strain or the HV strain (**Figure 8**). Expression of *PR5* could not be detected in a consistent way and was discarded for further analysis. *PR1* showed an early downregulation in plant roots infected either by the WV strain or the HV strain, but 1 day later its expression was 50 times higher in roots infected by the WV strain than in roots infected by the HV strain. This difference was progressively attenuated and disappeared in infected root crown at 5 dpi. The expression of the *ERF1* gene was similar in plants infected either by the WV strain or the HV strain. Contrary to the *PR1* expression pattern, the induction of the *ERF1* gene reached a maximum in roots at 1 dpi and then slowly decreased. *ERF2* was also induced at early stages of infection but it was significantly overexpressed in roots infected by the HV strain. Therefore, two main findings must be highlighted. First, SAR response is repressed at the beginning of the infection while the ET-mediated response is activated in plants inoculated either with the HV or WV strains. Second, SAR response is delayed in plants infected by the HV strain with respect to those inoculated with the WV strain, while the ET responsive factor *ERF2* is significantly induced.

### *FTF1* Expression in *PTFT1::GFP* Transformants

**FIGURE 9:**
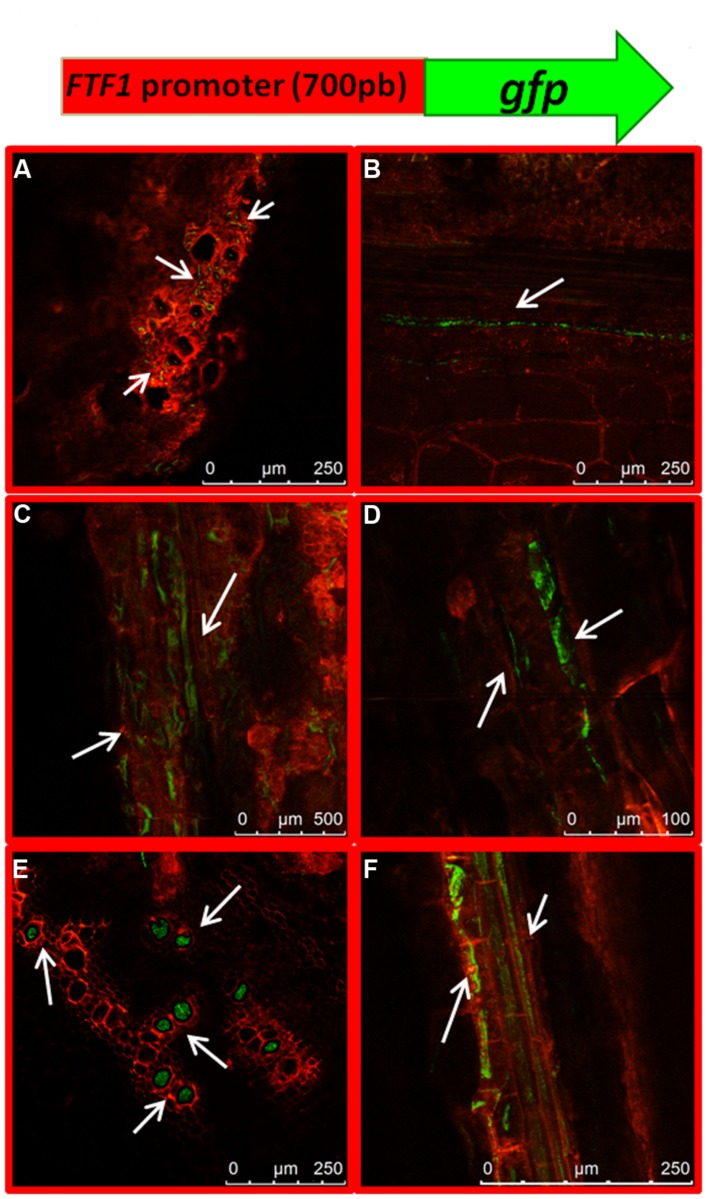
**Dynamics of plant colonization by a FOP-SP1 transformant expressing the *GFP* gene under the control of the *FTF1* promoter (*PFTF1::GFP*).** The transgene expressed in the transformant *PFTF1::GFP* is schematically depicted at the top of the figure. Arrows indicate mycelial growth as visualized in cross sections **(A,E)** and longitudinal sections **(B–D,F)** of root crown at 7 dpi **(A)** and hypocotyl at 10 dpi **(B)**, 14 dpi **(C,D)** and 21 dpi **(E,F)**.

The expression profile of the gene encoding the virulence factor FTF1 above described is in contrast with former reports which showed maximum expression in plant stem 24 h post inoculation (Ramos et al., 2007). In order to confirm our present observations, we obtained transformants of strain FOP-SP1 with a construct that included the *GFP* gene under the control of the *FTF1* promoter. The first observations of GFP-expressing mycelium in colonized plant tissues corresponded to the crown region at 7 dpi (**Figure 9A**) in accordance with a 10-fold overexpression of the gene. From this time GFP fluorescence was clearly visible inside the xylem vessels of hypocotyls at 10 dpi (**Figure 9B**) and 14 dpi (**Figures 9C,D**). Most of the xylem vessels in hypocotyls at 21 dpi were occluded by fungal mycelium (**Figures 9E,F**).

The expression pattern of *FTF1* in the confocal microscopy analysis matches the expression profile obtained in the RT-qPCR experiments, and confirms that upregulation of *FTF1* takes place in crown and hypocotyl from 7 dpi, under the present experimental conditions.

## Discussion

The vast majority of microscopy analysis of plant colonization by *F. oxysporum* have been related to the details of particular interactions (Bishop and Cooper, 1983; Czymmek et al., 2007; Nahalkova et al., 2008; Li et al., 2011), or the differences in the colonization of susceptible and resistant cultivars (Bishop and Cooper, 1983; Zvirin et al., 2010; Jiménez-Fernández et al., 2013; Lü et al., 2013). Almost no attention has been paid to the study of differences in plant colonization based on the virulence of the infecting strains. Most likely this is because of the scarce availability of strains able to infect the same host while displaying a range of virulence, which to our knowledge has only been shown in *F. oxysporum* f. sp. *phaseoli* (Alves-Santos et al., 2002a) and *F. oxysporum* f. sp. *dianthi* (Gómez-Lama Cabanás et al., 2012).

*Fusarium oxysporum* f. sp. *phaseoli* strains may be classified as weakly and highly virulent against *P. vulgaris* (Alves-Santos et al., 2002a) or as weakly, highly and supervirulent against *P. coccineus* (de Vega-Bartol et al., 2011). In a former report (García-Sánchez et al., 2010) we described two main differences in the plant colonization pattern of a HV strain (FOP-SP2) and a WV strain (FOP-SP8), the latter being slower in the rate of xylem colonization and with a reduced capability to colonize xylem vessels. With the aim to gain deeper insight into host colonization features, we have analyzed two more strains (FOP-SP1 and FOP-SP4) and used a hydroponics system which allows for easier access to plant roots for sectioning and confocal microscopy visualization.

Penetration of host roots by *F. oxysporum* is assumed to occur by direct penetration or through wounds (Nelson, 1981). In the absence of wounds, most reports show that the preferred zone of ingress is the root apex (Sarrocco et al., 2007; Jiménez-Fernández et al., 2013) or roothairs (Lagopodi et al., 2002). We did not observe differences between the HV and the WV strains with regards to the ingress area. Both strains showed a preference for the junctions of the lateral roots with the taproot, where most likely they can take advantage of discontinuities in the plant epidermis. The main difference observed at this stage between the HV and WV strains was the ability of the HV strain to reach the root vascular cylinder and quickly progress toward the root crown. The specialized dissemination of the HV strain inside the root is not related to a better growth rate as the total amount of fungal biomass in the root is very similar for both strains. The only differential expression detected in fungal genes corresponds to *SGE1* at 2 dpi and *RHR1* at 3 dpi, both upregulated in the WV strain. The genes which expression is exclusive to the HV strain (*FTF1*, *SIX1* and *SIX6*) do not show significant upregulation in root, indicating that their induction is not required for penetration nor for the colonization of this part of the plant. The absence of significant expression of *FTF1* in roots is in agreement with previous reports (Ramos et al., 2007). In contrast with these results, some differences related to virulence are found in the expression of plant defense genes. *PR1* is significantly downregulated during the first 24 h of infection by the HV strain, while the opposite occurs to the common bean homologue of *ERF2*, one of the genes involved in the ET/JA defense response (Fujimoto et al., 2000). Ethylene and JA biosyntheses are activated by a variety of forms of stress, including the biotic stress induced by pathogens, and both pathways converge in the transcriptional activation of *ERF1* (Lorenzo et al., 2002) what makes it a good marker to detect the activation of both phytohormones. The homologue of *ERF1* in common bean is overexpressed in plants infected either with the WV strain or the HV strain at 1 dpi. It is worth to note that this induction of *ERF1* is accompanied by the already mentioned downregulation of *PR1*, which occurs not only in plants infected by the HV strain, but also when they are infected by the WV strain, although to a lesser extent. Diverse analysis have revealed that SA, ET and JA pathways influence the outcome of the disease produced by *F. oxysporum* in *Arabidopsis* (reviewed in Berrocal-Lobo and Molina, 2008). Our results indicate that higher *PR1* gene and lower *ERF* genes expression during the first 2 days of root infection correlate with a higher rate of parenchyma colonization and a lower rate of vascular cylinder colonization (infection with the WV strain), and vice versa (infection with the HV strain).

Vascular pathogens are characterized by their ability to colonize the vascular system of the host. The collapse of the xylem vessels, caused by the accumulation of fungal mycelium and host defensive molecules, produces a drastic reduction in the transport of water to the aerial parts of the plant and finally its death. The architecture of the stem vascular system develops in the crown area where the primordial ring of xylem vessels is formed. We observed in this area the most drastic differences, either in the amount of fungal biomass and its distribution, between the strains analyzed. Expression of several genes in both strains at this time point seems to be important to sustain crown colonization, namely *FTF2*, *FNR1*, *EBR1*, *PacC* and *RHR1*. The upregulation of *FTF2* actually begins in the root, in the case of the WV strain, and it is surprising as former analyses carried out by our group suggested this gene was not involved in host infection (unpublished results). *FTF2* and *EBR1* are members of different gene family expansions characteristic of *F. oxysporum*. Both genes appear as single copies in the genomes of several *Fusarium spp*., but the paralogues *EBR2*, *EBR3*, *EBR4* and the copies of *FTF1* are present only in *F. oxysporum* (de Vega-Bartol, unpublished; Jonkers et al., 2013). However, the relative importance of the paralogues and the single copy genes are different. EBR1 is the most abundant transcript of the EBR family during host infection and its deletion results in a reduction of virulence, while the rest of the paralogues do not seem to have a role in host colonization (Jonkers et al., 2013). In the case of *FTF2*, the most abundant transcripts of the family during common bean infection are produced by the copies of *FTF1* and, although we have not yet analyzed a mutant deleted in *FTF2*, *FTF1* is exclusive to the HV strains of *F. oxysporum* f. sp. *phaseoli*.

The upregulation of *FNR1* and *RHR1* seems to be related to the adaptation of fungal nutrition to a new compromising environment. *FNR1* has been described to mediate the adaptation to nitrogen-poor conditions *in planta* (Divon et al., 2006), such as those potentially found inside the xylem vessels. The *A. nidulans* *rhaR* gene encodes a transcription factor that positively regulates the expression of both the enzymes that liberate L-rhamnose from complex plant substrates (alpha-L-rhamnosidases) as well as those involved in the assimilation of this sugar, and hence confers *A. nidulans* the ability to adapt its metabolism to the availability of L-rhamnose (Tamayo-Ramos et al., 2012; Pardo and Orejas, 2014). Interestingly, RNA-seq data obtained from the *Aspergillus* Genome database (AspGD) suggest that *rhaR* could be preferentially expressed under nitrogen limiting conditions. Therefore, it is conceivable that once the mycelium of *F. oxysporum* grows inside the vascular system, where the preferred sugars are absent, it must obtain other carbon sources from the cell wall components of the lignified xylem vessels. There is a paralogue of *RHR1* in *F. oxysporum*, named *RHR2*, which does not show significant changes in expression during the colonization process, neither in the WV strain nor the HV strain.

PacC mediates regulation of gene expression by ambient pH in fungi (Peñalva et al., 2008). It has been shown in *F. oxysporum* f. sp. *lycopersici* that *PacC* transcript levels rise when the fungus is grown in alkaline conditions (Caracuel et al., 2003). Here we show that *PacC* transcript levels increase at 7 dpi, in coincidence with the beginning of the colonization of the hypocotyl xylem vessels. This suggests that ambient pH inside the vessels is alkaline and that genes expressed under alkaline conditions are required for their colonization by either HV or WV strains.

The highest levels of gene expression were detected in the hypocotyl at 14 dpi and correspond to *FTF1*, which is unique to the HV strains, and the effector encoding genes *SIX1* and *SIX6*. At that time, observations of plants infected with the HV strain show that growth is restricted to the xylem vessels, thus indicating that *FTF1*, *SIX1* and *SIX6* are expressed during colonization of the vascular system. SIX1 and SIX6 are recognized as genuine effectors, as they contribute to the general virulence of *F. oxysporum* f. sp. *lycopersici* (Rep et al., 2004; Gawehns et al., 2014). Homologues of these genes have been found in other formae speciales like *F. oxysporum* f. sp. *cubense* (Niño-Sánchez, unpublished) and *F. oxysporum* f. sp. *phaseoli* (this work), although only the copies present in the HV strain are expressed during infection. It cannot be ruled out that the *SIX1* and *SIX6* alleles in the genome of the WV strain are not functional. Alternatively, the expression of *SIX1* and *SIX6* might need a transcription activator not present in the WV strain, such as *FTF1*. The expression of *FTF1*, *SIX1* and *SIX6*, was detected at low levels in root 1 dpi, in accordance with the former results obtained for *SIX1* during tomato infection (van der Does et al., 2008). Upregulation of the three genes begins in the root crown 5 dpi, matching the accumulation of mycelium in this area and the beginning of massive colonization of xylem vessels.

The classical view of plant colonization by fungal vascular wilt pathogens assumes that, after penetration, the fungi colonize the cortical cells and then hyphae migrate intercellularly toward the vascular parenchyma cells and invade xylem vessels. Once the xylem is reached, the mycelium is mostly confined inside the vessels, until the necrosis of host tissues allows for general colonization (Yadeta and Thomma, 2013). In this work we have shown that a high level of virulence correlates with a high ability to quickly infect and colonize the plant vascular system, with almost no colonization of the root cortex, and that WV strains are efficient colonizers of host tissues before they become necrotized. Also, our data suggest a correlation between virulence and the expression of some components exclusive of the genome of HV strains, such as the FTF1 transcription factor, and other components that, although not exclusive, are only expressed in HV strains, such as the effectors SIX1 and SIX6. *FTF1* paralogs are part of the unique sequences found in the genome of *F. oxysporum*, designated as lineage specific (Ma et al., 2010). It has been proposed that *F. oxysporum* lineage specific sequences located in dispensable chromosomes are the basis of host specialization and could explain the polyphyletic origins of most formae speciales (Ma et al., 2010). Our results show that they also contribute to explain *F. oxysporum* virulence.

## Conflict of Interest Statement

The authors declare that the research was conducted in the absence of any commercial or financial relationships that could be construed as a potential conflict of interest.
